# A plea for taking all available clinical information into account when assessing the predictive value of omics data

**DOI:** 10.1186/s12874-019-0802-0

**Published:** 2019-07-24

**Authors:** Alexander Volkmann, Riccardo De Bin, Willi Sauerbrei, Anne-Laure Boulesteix

**Affiliations:** 1 Institute for Medical Information Processing, Biometry and Epidemiology, University of Munich, Marchioninistr. 15, Munich, 81377 Germany; 2 Department of Mathematics, University of Oslo, Moltke Moes vei 35, Oslo, 0851 Norway; 3 Institute of Medical Biometry and Statistics, Faculty of Medicine and Medical Center, University of Freiburg, Stefan-Meier-Straße 26, Freiburg, 79104 Germany; 4 Chair of Statistics, School of Business and Economics, Humboldt-Universität zu Berlin, Spandauer Straße 1, Berlin, 10178 Germany

**Keywords:** Data integration, Cox regression, Model building

## Abstract

**Background:**

Omics data can be very informative in survival analysis and may improve the prognostic ability of classical models based on clinical risk factors for various diseases, for example breast cancer. Recent research has focused on integrating omics and clinical data, yet has often ignored the need for appropriate model building for clinical variables. Medical literature on classical prognostic scores, as well as biostatistical literature on appropriate model selection strategies for low dimensional (clinical) data, are often ignored in the context of omics research. The goal of this paper is to fill this methodological gap by investigating the added predictive value of gene expression data for models using varying amounts of clinical information.

**Methods:**

We analyze two data sets from the field of survival prognosis of breast cancer patients. First, we construct several proportional hazards prediction models using varying amounts of clinical information based on established medical knowledge. These models are then used as a starting point (i.e. included as a clinical offset) for identifying informative gene expression variables using resampling procedures and penalized regression approaches (model based boosting and the LASSO). In order to assess the added predictive value of the gene signatures, measures of prediction accuracy and separation are examined on a validation data set for the clinical models and the models that combine the two sources of information.

**Results:**

For one data set, we do not find any substantial added predictive value of the omics data when compared to clinical models. On the second data set, we identify a noticeable added predictive value, however only for scenarios where little or no clinical information is included in the modeling process. We find that including more clinical information can lead to a smaller number of selected omics predictors.

**Conclusions:**

New research using omics data should include all available established medical knowledge in order to allow an adequate evaluation of the added predictive value of omics data. Including all relevant clinical information in the analysis might also lead to more parsimonious models. The developed procedure to assess the predictive value of the omics data can be readily applied to other scenarios.

**Electronic supplementary material:**

The online version of this article (10.1186/s12874-019-0802-0) contains supplementary material, which is available to authorized users.

## Background

The abundance of information contained in omics data seems like a feast for researchers. The sheer amount of candidate variables almost guarantees some positive findings, whatever the research question is [1]. In many cases, the apparent good prediction performance of molecular signatures to predict a clinical outcome is due to methodological flaws [2]. In other cases, the identified signatures may not be much more than surrogates for clinical variables [3]. Even though it can occasionally be of interest to identify possible omics representatives for clinical data, researchers are typically interested in *additional* insights rather than replacing classical clinical predictors with omics scores. In most cases, clinical variables are routinely collected and thus available by default without additional costs. There is thus an essential asymmetry between clinical and omics variables: in terms of prediction, omics data are useful only if they can increase prediction performance compared to models using clinical data only. When building omics-based prediction models, the hope is to capture aspects of the prediction problem that are not already captured by clinical variables. In this article we will focus on this (very common) situation. Note that clinical variables are sometimes used in practice to define subgroups of patients which are then further investigated separately, using omics data. However, this case is not considered here.

In light of these considerations, it becomes clear that it is of utmost importance in omics model building to not only appropriately deal with the high dimensionality of omics data but also to adjust for already existing knowledge in the form of clinical information. The integration of clinical data, dense in information, and omics data has attracted more and more attention in recent years, especially in the field of predictive^1^ modeling of clinical outcomes [4, 5]. For two example data sets De Bin et al. [6] systematically compare different strategies for combining omics and clinical data when modeling survival outcomes. Vazquez et al. [7] apply a Bayesian approach to incorporate clinical and multiomic data into prediction models. Dimitrieva et al. [8] compare the predictive powers of clinical to expression and methylation data on survival and find that only the integration of molecular and clinical variables results in improved predictions.

Yet even studies that do include clinical information often put considerably more effort into modeling the omics part of the predictor than into modeling the clinical part. Simple procedures, such as least squares regression, cannot be applied to handle high-dimensional data: this is probably why researchers focus their attention on this tricky part of the modeling process, while comparatively ignoring the modeling of clinical data, which they consider to be less problematic. Accounting for clinical variables naively (for example, by adjusting for a well-known marker without considering further potential variables) is better than completely ignoring them. However, if the available clinical variables are not sufficiently incorporated into the model, the added predictive value of omics data might be overestimated. In other words, omics data might appear more useful than they actually are, as a result of not fully exploiting the potential of clinical variables.

For example, the prediction of the survival of breast cancer patients, first based on clinical variables and then on omics data, has been an important research topic for years. About 35 years ago the Nottingham Prognostic Index (NPI) was proposed [9]. The NPI was validated in many independent data sets and is a well accepted tool to predict recurrence free and overall survival time for patients with primary breast cancer [10]. Using the full information from standard clinical data Winzer et al. [11] propose an extended version with improved prognostic ability. They recommend using this extended version as a benchmark to assess the added value of new information in this area.

The goal of this paper is two-fold. Firstly, we demonstrate empirically, based on two applications to breast cancer data, that the apparent added predictive value of omics data for prognostic purposes depends on the considered clinical model. Our conjecture is that the observed increase in prediction performance yielded by omics data diminishes when including more information from clinical variables in the model. Secondly, we describe simple procedures to take more clinical information into account with different levels of clinical model complexity when building an omics-based model. This strategy may be used as a suitable pipeline for future research projects on the usefulness of omics data.

For these purposes, we use results from Winzer et al. [11] to assess the added predictive ability of models with omics variables that were constructed on various levels of clinical information. For each of these levels, omics variables are selected via different variable selection methods and combined with the clinical variables into a Cox proportional hazards model [12] using a training data set. The resulting models are evaluated on an independent validation data set and compared to the corresponding models that only include the clinical information of the respective level. In this way, the added predictive value of the omics variables can be investigated for models using a different amount of clinical information. Data and code are available online so that all analyses are reproducible.

The paper is structured as follows: first, the two considered breast cancer data sets are introduced and the study design, as well as the implemented methods, are described. Subsequently, the results are presented and discussed.

## Methods

### Data

This study uses two different data sets to evaluate the added predictive value of omics variables in the field of breast cancer. For a data set to be suitable for the study, it has to contain omics data (in the form of gene expression data) as well as a decent amount of prognostic clinical information and the survival status of the patients.

#### Hatzis data

The first data set contains information on 508 newly diagnosed ERBB2-negative breast cancer patients treated with neoadjuvant chemotherapy. Hatzis et al. [13] collected information from 2000 to 2010 for their study on a predictor of response to and survival after chemotherapy. The data is available online from the GEO database [14] under the accession number GSE25066. It includes clinical variables as well as processed gene expression microarray data for 22,283 probe sets, separated into two independent training and validation data sets containing *n*=310 and *n*=198 observations, respectively. The censored response used in this study is the distant relapse free survival time (the time interval between the initial diagnosis and either the diagnosis of distant metastasis or death), with 66 and 45 events in the training and validation data set, respectively. Missing values in the clinical variables of the tumor grade and estrogen receptor status of a patient’s tumor are imputed using the clinical information considered in this project plus additional information on the progesterone status of a patient via a single imputation within the multivariate imputation procedure by chained equations algorithm as implemented in the R add-on package mice [15]. A small constant (1e-05) was added to the survival time of a patient who experienced the event at day 0, to include this observation in the modeling process. A detailed description of the clinical variables included in the data set can be found in Table I of the original paper. In contrast to the original study and similarly to De Bin et al. [6], we use all available information for nodal status and tumor size. Note that two patients in the training data and one patient in the validation data with T0 tumors are collapsed with the T1 group. In the most complex model we include the continuous variable age. From many studies it is well known that this effect is non-linear, which is why we include it adopting the best fitting fractional polynomial function of degree 2 [16]. Using the routine implemented in the R package mfp [17] and forcing the program to include all variables by setting their p-value for exclusion to 1, the FP2 function with power terms (-2, -2) is selected in the training data.

#### GDC data

The second data set is made publicly available by the Genomic Data Commons (GDC) Data Portal [18] which provides harmonized cancer data sets for over 25 different cancer types. The information on breast invasive carcinoma stems from The Cancer Genome Atlas (TCGA) [19]. As the database is still being further developed, we work with the data release 10.0, which provides information on breast cancer patients with clinical and follow-up data as well as gene expressions for 56,963 genes. Starting with 1097 patients with existing gene expression information, we first exclude patients who are either (i) male, (ii) have received neoadjuvant therapy (which was uncommon at the time of treatment), or (iii) whose tumor has spread to a different part of the body. We also exclude those patients with missing information on survival. In order to analyze the disease free survival time, we combine information on follow up and new tumor events to construct the target variable. Missing values for clinical variables are imputed as described for the Hatzis data set and similar transformations of the survival times are carried out. In this data set, age is included as a (3,3) fractional polynomial as selected using the mfp package (see above). This results in a baseline data set of 1039 breast cancer patients either alive and without recurring cancer (*n*_*c*_= 856) or with new tumor event or dead (*n*_*e*_=183) with median survival time of 783 days. A detailed description of the clinical data can be found in Additional file 1: Section A. For this set of patients the omics data contain a considerable amount of very low gene expressions, which is why we restrict the omics variables to be analyzed to those that show expression values unequal to 0 for at least half of the patients. Consequently, only 30,913 different gene expression variables are included in the following analysis. Finally, we randomly split the data into a training set that consists of two thirds of the data and a validation set, using the disease free survival status as a stratifying variable to ensure that the proportion of censored observations is the same in the training and validation sets. This means that the resulting training data set contains 692 observations, 122 of which are informative (events), whereas the validation data set comprises the remaining 347 observations (61 events).

Note that there is a subtle difference between the two data sets: while the training data provided by Hatzis et al. [13] consists entirely of patients that received a neoadjuvant chemotherapy regimen, the validation data set also contains 15 patients that received an entirely adjuvant chemotherapy. Unfortunately, we were not able to identify those patients to further homogenize the data base. However, it is unlikely that this difference in treatment has a relevant influence on the results of our investigation. For the GDC data, however, we explicitly exclude all patients that received neoadjuvant therapy to align the data sets used for model building and model evaluation with respect to timing of chemotherapy.

### Model building

A popular way of modeling the effect of variables on survival time consists in applying extensions or variants of the multivariate Cox regression model. Even though other methods are available and might yield better results in some cases, we deliberately restrict our modeling process to this approach. One advantage of this is the resemblance to well-known linear regression techniques (in contrast to nonparametric procedures such as random forest [20]); another is the already existing research on how to combine low-dimensional clinical and high-dimensional omics data for these methods (e.g. [6, 21]). Moreover, applying a modeling approach that belongs to the standard tool kit of researchers might convey the message of this paper better than a more refined modeling strategy specifically adapted to the particular data situation at hand.

Thus, we model the hazard *λ*(*t*|*Z*_1_,...,*Z*_*q*_,*X*_1_,...,*X*_*p*_), which is the instantaneous failure rate, depending on the clinical variables *Z*_1_,...,*Z*_*q*_, with *q*<*n*, and the omics variables *X*_1_,...,*X*_*p*_, with *p*≫*n*. The constructed models take the following form: the hazard is modeled as the product of the baseline hazard function *λ*_0_(*t*) with the exponential of a linear predictor *η* consisting of the clinical variables (e.g. tumor size or nodal status) and omics variables (e.g. the expression levels of different genes):

1 $$ \begin{aligned}  &\lambda(t\mid Z_{1},...,Z_{q}, X_{1},..., X_{p}) = \lambda_{0}(t) \cdot \text{exp}(\eta) \\ &\text{with} \ \eta = \gamma_{1} Z_{1} +... + \gamma_{q} Z_{q} + \beta_{1} X_{1} +... + \beta_{p} X_{p}, \end{aligned}  $$

where *γ*_1_,...,*γ*_*q*_ and *β*_1_,...*β*_*p*_ are the regression coefficients of the clinical and omics variables, respectively. Note that it is clear from Eq. (1) that we assume all variables to have a linear effect (except for age, which enters the model in its transformed form with power terms (-2,-2) in the GDC and (-3,-3) in the Hatzis data, respectively) and only consider models with main effects, as usual in this context.

The challenge with omics data in particular is the surplus of variables *p* over the number of observations *n* (*n*≪*p* problem). As a result, classical maximum-likelihood methods fail, which is why omics data typically call for dimension reduction, regularization approaches or variable selection techniques. In this study we apply two popular methods of regularization that eventually lead to variable selection and provide interpretable prediction models. We use a clinical model with variables based on medical knowledge as a starting point to summarize clinical information (i.e. a model of form Eq. (1) with *β*_1_=...=*β*_*p*_=0). We then use this clinical model as an offset and identify possible informative omics variables using regularized Cox models with regularization only on the omics variables *X*_1_,...,*X*_*s*_, as explained in more detail in the next paragraph.

In order to fully exploit the predictive value of each level of clinical information, we start by fitting a Cox regression on the clinical variables only. The resulting estimates $\hat {\gamma }_{1}^{of},..., \hat {\gamma }_{q}^{of}$ of the clinical coefficients enable us to use the linear predictor $\hat {\eta }^{of} = \hat {\gamma }_{1}^{of} Z_{1} +... \hat {\gamma }_{q}^{of} Z_{q} $ as an offset in the omics variable selection procedure (see “Selection of omics variables” section). This scenario corresponds to the strategy 2 discussed in Boulesteix and Sauerbrei [21], where the regression coefficients *γ*_1_,...,*γ*_*q*_ of Eq. (1) are fixed at the values $\hat {\gamma }_{1}^{of},..., \hat {\gamma }_{q}^{of}$ derived from the clinical model. As a result, the coefficients *β*_1_,...,*β*_*p*_ are estimated only on the residuals of the clinical prediction model, whereas the estimation of *γ*_1_,...,*γ*_*q*_ is not affected at all by the omics variables. Boulesteix and Sauerbrei [21] state, however, that this strategy might be suboptimal in terms of prediction accuracy and it also might be susceptible to potential bias caused by building the clinical model on the same data that are used for the estimation of the final predictive model. Although the omics part is fitted on the residuals of the clinical model (and, therefore, the information included in the data is not used twice), the residuals’ variability depends on the clinical model fitting process. Nonetheless, we think that this approach might be able to reflect the added predictive value of omics variables when compared to an already existing clinical model.

**Fig. 1:**
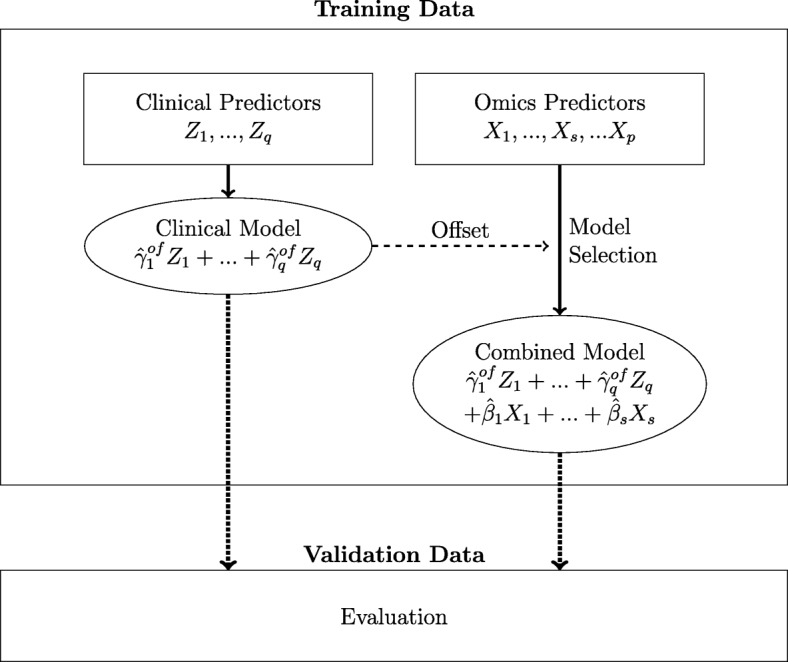
Model building process. This leads to the two predictive models which are evaluated on the validation data. Note that, depending on the considered scenario, the clinical predictor may be zero (in case no clinical variables are considered), include clinical variables selected by general knowledge, and/or include variables which are transformed as a result of MFP analysis

We therefore build two Cox regression models for each level of clinical information: a purely clinical model and the so called combined model. Figure 1 illustrates the model building process that leads to these models. A notable exception is the level of no clinical information at all, where there is evidently no clinical offset for the variable selection of omics variables. Consequently, the variable selection and model building strategy is applied to the omics data without specifying an offset. A single model with linear omics predictor *η*^*null*^=*β*_1_*X*_1_+...+*β*_*s*_*X*_*s*_ is therefore obtained. We include this level in our study to further emphasize the need for adequate consideration of clinical variables.

### Levels of clinical information

Based on years of experience in breast cancer research of one of the authors and in particular the results of Winzer et al. [11], we propose four models containing differing amounts of clinical information, which are summarized in Table 1. These models serve as reasonable starting points for predicting the survival of breast cancer patients and are loosely based on the Nottingham Prognostic Index but adapted to the available data. The different levels of clinical information are designed in such a manner that the most accepted prognostic factor, nodal status, is included first followed by the number of positive nodes. In the following models we add tumor size and grade, the other two components from the Nottingham Prognostic Index. The prognostic role of estrogen receptor and age has been discussed for a long time. Based on the results of Winzer et al. [11] we add estrogen and finally we add the continuous variable age, for which we determine the functional relationship with the fractional polynomials (FP) approach. Consequently, the set of clinical variables *Z*_1_,...,*Z*_*q*_, as defined above, contains age transformed according to a member of the FP family.

**Table 1:** Levels of clinical information

Model	nodal status	nodal status	tumor size	tumor grade	estrogen receptor	age
	(N0/N+)	(N0, N1, N2, N3)	(T0_1, T2, T3, T4)	(1,2,3)	(yes/no)	(FP)
M0	-	-	-	-	-	-
M1	X	-	-	-	-	-
M2	-	X	X	[X]	-	-
M3	-	X	X	[X]	X	-
M4	-	X	X	[X]	X	X

The square brackets indicate variables that are only available for the Hatzis data set

A detailed summary of the levels of clinical information included in the modeling process can be found in Table 1. The final model M4 contains information about the nodal status (4 categories), tumor size (4 categories), tumor grade (3 categories), estrogen receptor status (dichotomous) and age (continuous). As a result, all models of level M4 (purely clinical and combined) include the five clinical variables *Z*_*nodalstatus*_,...,*Z*_*age*_ selected based on previous clinical knowledge. While the variable selection procedures decide on which omics variables to include in the combined model, there is no further selection of clinical variables even if they show no significant effect in the purely clinical model. Note that the information on tumor grade is not available for the GDC data set and could therefore not be included in the modeling process which is indicated by square brackets.

To contrast the results with the scenario where clinical information is completely ignored, we also consider a clinical null model (M0) that does not contain any clinical variables at all. This model is therefore handled somewhat differently in the model building process (as described above).

### Selection of omics variables

For each level of clinical information, we re-apply the variable selection procedures in order to identify relevant omics variables for distant relapse free survival. We turn to well-known and widely used methods that are applicable to Cox regression: the Least Absolute Shrinkage and Selection Operator (LASSO) [22] and model based boosting [23]. Each variable selection procedure can result in a different set of selected omics variables that determines the model based on the training data and provides the corresponding predictor.

The LASSO is a penalized regression approach which uses a *L*_1_ penalty. The presence of this penalty allows the handling of situations where *n*≪*p* and forces some regression coefficients to be exactly 0, i.e. leads to variable selection. We modify the standard Lagrange multiplier version of the underlying optimization problem by including the estimators of the clinical model as an offset, i.e. we estimate ***β*** as

2 $$\begin{array}{*{20}l} \boldsymbol{\hat{\beta}} = \arg\,\max \{\ell(\boldsymbol{\hat{\gamma}^{of}}, \boldsymbol{\beta}) - \lambda \Vert\boldsymbol{\beta}\Vert_{1}\}, \end{array} $$

with $\ell (\boldsymbol {\hat {\gamma }^{of}}, \boldsymbol {\beta })$ the partial log-likelihood of model (1) where the parameters in the parameter vector ***γ***=(*γ*_1_,..,*γ*_*q*_)^⊤^ are set to the estimates of the clinical model $\boldsymbol {\hat {\gamma }}^{of} = (\hat {\gamma }_{1}^{of},.., \hat {\gamma }_{q}^{of})^{\top }$ and only the parameter vector ***β***=(*β*_1_,...,*β*_*p*_)^⊤^ is to be estimated. A fundamental challenge of the LASSO approach is choosing the regularization parameter $\lambda \in \mathbb {R}^{+}$. Here, this is done by determining the value of *λ* that results in the minimal mean cross-validated error based on the deviance on 10-fold cross-validation.

The boosting approach for regression is slightly different, and can be seen as a forward stagewise procedure which, starting from the null model, iteratively updates the regression coefficients through a penalized estimator (base-learner). The goal is to minimize a loss function, in general the negative log-likelihood (in our case, i.e. for Cox regression, the negative partial log-likelihood). In its componentwise version, each regression coefficient is updated separately: when the algorithm is stopped sufficiently early, the boosting approach assures variable selection (the regression coefficients related to irrelevant variables remain 0) and shrinkage (due to the updating process stopping early). Importantly, the componentwise version of boosting allows working in the *n*≪*p* framework. The stopping criterion is determined using 25-fold bootstrap iterations. The aforementioned offset strategy can be easily implemented within the boosting algorithm. It is sufficient to start from the clinical model (as an offset) instead of the null model [24].

Both variable selection approaches are readily available and implemented in the R packages glmnet [25] and mboost [26], respectively. The level of regularization is determined using the default option of the corresponding implementation.

### Model evaluation

Evaluating a survival prediction model is not straightforward. This can be seen from the wide range of literature that has dealt with this issue (e.g. [27, 28]). Cook [29] specifically points out that no single measure exists that could serve as an “ideal method to evaluate the added [predictive] value of new biomarkers. Several methods evaluate different dimensions of performance and should be considered [...]". Along the lines of De Bin et al. [6], we restrict ourselves to the assessment of the predictive ability of a model via two of the most popular measures: the concordance index (C-index) [30, 31] and the integrated Brier Score (IBS) [32].

The C-index indicates the ability of a model to separate the survival curves of groups with differing risks by quantifying rank-correlation between predicted and observed outcomes. The measure usually takes values between 0.5 and 1 which mark no discrimination and perfect separation of observations with different outcomes, respectively. Here, we use an alternative version of the original C-index by Harrell et al. [30] that takes censoring into account [31]. Note that for the clinical model M0, the C-index is not defined as the model is not able to assign different risks to any two subjects [31]. In these cases, we set the C-index to the value 0.5 (corresponding to a random guess) to allow comparisons.

The IBS, on the other hand, not only considers the discrimination of a model but also the calibration, which is the similarity between the actual and the predicted outcomes [32]. The measure is based on the Brier score, which builds on the predicted time-dependent survival probability [33]. It should be stressed that while a higher C-index is associated with a better discriminative ability, the lower the IBS the better the predictive ability of a model. These measures are applied both to the training and validation data sets. One has to keep in mind that an evaluation on the training data set yields results that are too optimistic and therefore cannot be trusted for model comparison [34]. Both C-index and IBS are implemented using the R package pec [35].

For each level of clinical information, we identify the added predictive value of the omics variables by subtracting the concordance index of the purely clinical model from the respective index of the combined model. Positive differences indicate an added predictive value for the gene expression data, whereas negative differences imply that the purely clinical models perform better. Due to the differing orientation of the IBS (smaller values show better predictive ability) we subtract the values of the combined models from the purely clinical models to facilitate interpretation. In this way, we compare the added predictive value over different amounts of clinical data and different evaluation criteria.

Furthermore, we do not want to base our conclusions on one model building run only. In order to take the high instability of omics data modeling into account, we also conduct a simple subsampling analysis: First, a subset of 80% of the observations in the training data are randomly drawn without replacement. Then, the model building process as described above is applied to this subsample. The evaluation, however, is based on the full validation data set to ensure comparability between subsamples. This procedure is repeated 100 times; thus, we get an idea of the variability in added predictive values for each level of clinical information.

## Results

We restrict the discussion of the results to the analysis of the boosting method as the results of the LASSO are very similar (see Additional file 1: Section B). The code to reproduce the analysis is available in the online supplement, Additional file 2. We first focus on the evaluation of the IBS and the C-index on the Hatzis data, then on the GDC data.

### Hatzis data set

In this section, we summarize the main results of the evaluation on the validation data set derived from models using the whole sample, as well as the subsampling scheme of the training data. For both measures, IBS and C-index, we find higher differences between the clinical and the combined model for lower levels of clinical information. When more and more clinical information is included in the modeling process, the differences between the models seem to vanish. This is the case for models derived from the whole, as well as from the subsampled, training data. We will now have a closer look at the different results.

#### IBS

**Fig. 2:**
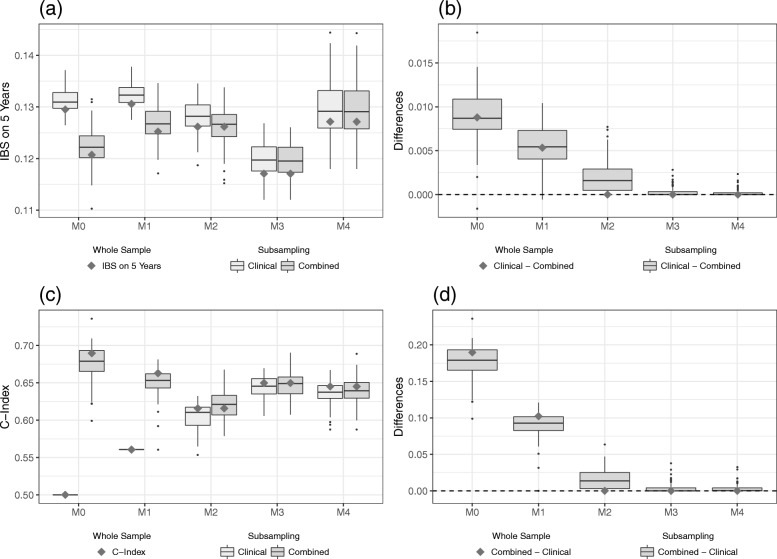
Results on the validation part of Hatzis data. IBS values (**a**) for the clinical and the combined models and the differences (**b**) between the models as evaluated on the validation data set. C-index values (**c**) for the clinical and the combined models and the differences (**d**) between the models as evaluated on the validation data set. The gray squares indicate the values of the models developed on the whole training data set, whereas the boxplots indicate the values obtained from the subsampling analysis

Figure 2a shows the calculated IBS values for both the models developed on the whole training data set (gray diamonds) and the subsampling scheme (boxplots), the lighter gray standing for the purely clinical models and the darker shade for the combined models. In theory, including more clinical information into the modeling process should result in lower IBS values for both clinical and combined models, indicating an improvement of the predictive ability, given the clinical information is indeed useful and does not lead to overfitting. However, we do not find such a straightforward relationship for this data set. In fact, model M0 seems to have a slightly higher predictive ability than model M1 (i.e. overall lower IBS values). This suggests that the Kaplan-Meier estimate derived on the training data can better predict the survival times on the validation data than the model that takes at least some clinical information into account. Nevertheless, when only models M1 to M3 are considered, the expected downwards trend can be discerned. Model M4, on the other hand, shows signs of potential overfitting as its IBS values are overall higher than most values of the models M2 and M3. Additionally, the dispersion of IBS values for model M4 is rather wide, which reflects that the metric variable “age” (in its FP2 transformation) renders the models quite flexible and thus susceptible to overfitting. Overall, model M3 seems to have the highest predictive ability and yields lower IBS values than the combined model M0, especially. This suggests that the clinical information available for this data set can have a positive influence on the predictive ability of survival models.

It is also apparent from Fig. 2a that the IBS values between the purely clinical and the combined model differ considerably for M0 and M1. Figure 2b shows the differences between the two types of models for each level of clinical information. The difference (clinical model minus combined model) was chosen in such a way that positive values reflect an added predictive value of the gene expression data. We see a pronounced added predictive value especially for the model with no clinical information at all. This added predictive value, however, seems to vanish as more and more clinical information is included in the modeling process. Particularly, for model M3 we do not see much of a difference between the purely clinical model and the combined model. In fact, starting with clinical model M2, the boosting algorithm no longer selects any omics predictors on the whole data set. Note that almost no difference is negative, which indicates that including omics variables in the modeling process does not worsen the predictive performance on independent data. We conclude that the gene expression data have predictive value as measured by the IBS, yet the *added* value seems to strongly decrease with increasing levels of clinical information included.

#### C-index

We now turn to the evaluation of the predictive ability using the C-index. Figure 2c contains the calculated C-index values for the validation data set of the Hatzis data in the already familiar representation. Keep in mind that there is one important difference: for the C-index, higher values indicate an increase in predictive ability. Note that the C-index, which measures the discrimination ability, can be interpreted as the probability that two randomly drawn patients are correctly ordered by the model in terms of survival time. Since the purely clinical model M1 contains only a binary variable, the predictor only takes two different values (depending whether the binary variable equals 0 or 1). Thus, the C-index values are identical for all resampling iterations provided the fitted coefficient of the binary variable has the same sign. Furthermore, the purely clinical model M0 is set to have a C-index of 0.5, corresponding to the predictive ability of a random guess. Just as for the IBS, we do not find a simple (in this case increasing) relationship between the level of clinical information and the overall levels of C-index values. Figure 2c again shows a considerable discrepancy between the purely clinical and combined models, for the models with little clinical information. For higher levels of clinical information, however, this difference diminishes once more. Again, the model M4 shows signs of potential overfitting compared to model M3 (albeit less pronounced than for the IBS) as the C-index values tend to be lower. A notable discrepancy between IBS and C-index is that the IBS identifies the model M3 to have the highest predictive ability whereas the C-index suggests that a model without adjusting for available clinical information yields the best results on the validation data set. Although this might seem contradictory, keep in mind that the IBS and the C-index measure different aspects of the prediction performance of a model.

In order to assess the added predictive value of the omics data using the C-index, we look again at the differences between the purely clinical and the combined models for each level of clinical information. Note that we consider the reverse difference (the C-index of the combined model minus the C-index of the clinical model) to facilitate comparisons between IBS and C-index. The differences of the C-index (Fig. 2d) look quite similar to Fig. 2b. We observe a considerable added predictive value for levels of low clinical information, that, however, vanishes as we include more and more clinical variables into the modeling process. Keep in mind that in this case the models M0 and M1, which incorporate omics data, also yield an overall higher predictive ability compared to the models with more clinical information. Considering that most differences are again positive, these results support the conclusion that for the Hatzis data set the omics variables do not harm the predictive ability. Additional file 1: Section C includes a table to summarize the results using the (median) values of the measures IBS and C-index.

**Fig. 3:**
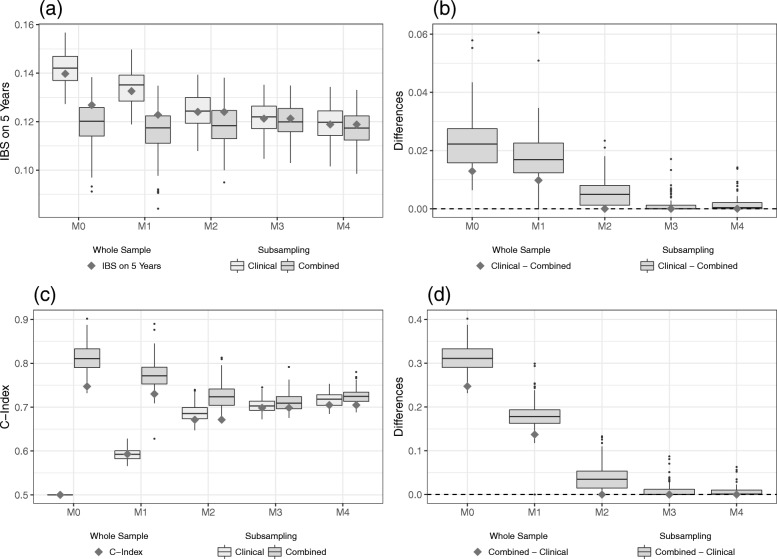
Results on the training part of Hatzis data. IBS values (**a**) for the clinical and the combined models and the differences (**b**) between the models as evaluated on the training data set. C-index values (**c**) for the clinical and the combined models and the differences (**d**) between the models as evaluated on the training data set. The gray squares indicate the values of the models developed on the whole training data set, whereas the boxplots indicate the values obtained from the subsampling analysis

It is also possible to evaluate the predictive ability on the training data. However, this is not recommended as this approach only considers the apparent error (also often denoted as training error in the literature) and thus overestimates the predictive ability. When evaluating the predictive ability using the training data, the Hatzis data show a downwards trend for the IBS values only for the purely clinical model (Fig. 3a). Incorporating more clinical information does not seem to noticeably improve the IBS score of the combined models. Consequently, we find a decreasing added predictive value for higher levels of clinical information on the training data (Fig. 3b). For the C-index we find a similar pattern as on the validation data but even more pronounced: by including more clinical information, the combined models that incorporate the omics data drop in predictive ability (Fig. 3c). The differences between the clinical and the combined models as given in Fig. 3d, thus show an apparent considerable added predictive value of the omics data which decreases with the amount of clinical information considered. Note that when using the training data to validate the models we can confirm that the poor performance of model M4 on the validation data is due to overfitting since it performs well on the training data for both the IBS and the C-index.

### GDC data set

We will now turn our attention to the GDC data set. In contrast to the Hatzis data set, we find that the differences between the clinical and the combined models are generally low and that omics information has hardly any effect. In the following, the results are discussed in more detail.

#### IBS

**Fig. 4:**
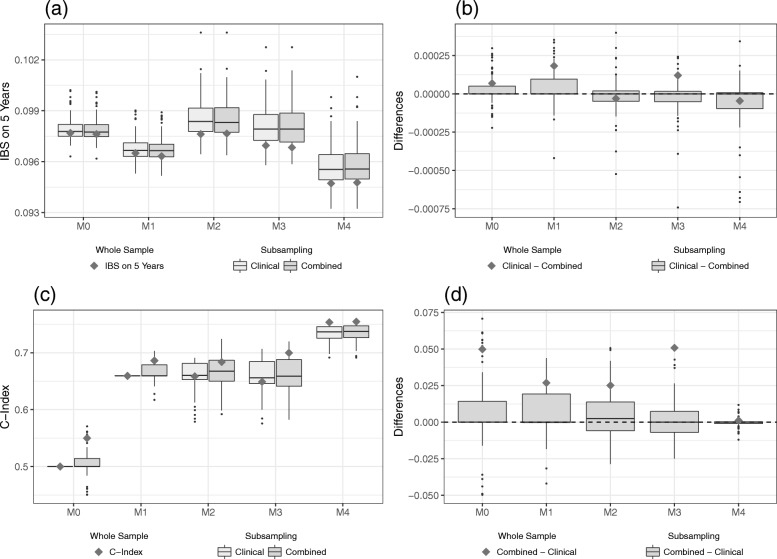
Results on the validation part of GDC data. IBS values (**a**) for the clinical and the combined models and the differences (**b**) between the models as evaluated on the validation data set. C-index values (**c**) for the clinical and the combined models and the differences (**d**) between the models as evaluated on the validation data set. The gray squares indicate the values of the models developed on the whole training data set, whereas the boxplots indicate the values obtained from the subsampling analysis. Two negative differences for model M4 are removed in (**b**)

Figure 4a shows the IBS values calculated on the validation data set. Here, we can identify two possible downwards trends (M0 - M1 and M2 - M4) indicating a better predictive ability for higher levels of clinical information. However, the increase in IBS values between the models M1 and M2 is somewhat unexpected. In contrast to the Hatzis data set, which shows signs of overfitting for models that include the variable age, model M4 now yields the overall lowest IBS values. Another notable difference is that the purely clinical and the combined models seem to differ less, even for models with little clinical information. The results suggest that on this data set, the level of clinical information considered in the modeling process has more influence on the predictive ability as measured by the IBS than the inclusion of omics variables.

As a result, we find that the differences in IBS values between the two types of models are on a smaller scale than for the Hatzis data set (see Fig. 4b). Moreover, there is a considerable number of negative differences suggesting that incorporating omics data may worsen the predictive ability of models. While all boxplots show a skewed distribution of the differences, it is interesting to note that for low levels of clinical information the boxplots are skewed to the right (meaning a broader range between median and upper quartile), whereas the reverse is true for more clinical information. As the median for all levels of clinical information is zero, this suggests that models with higher levels of clinical information more often suffer when including omics data, while models with little clinical information might benefit.

#### C-index

Just as for the IBS, the results with the C-index as evaluation measure suggest greater differences between the levels of clinical information than between the clinical and the combined models. Figure 4c shows a considerable increase in C-index values for models that adjust for clinical information compared to the model M0. It is worth noting that many values of the combined model for M0 lie at 0.5 or below, indicating a predictive ability of a random guess or worse, respectively. This might be an indication that the omics variables of this data set do not have much predictive information to begin with. Further inclusion of clinical information, however, does not seem to considerably boost the predictive ability, which results in a more or less steady level of C-index values for models M1 to M3. Only by adding the variable age to the modeling process, do we see a rise in C-index values. As a result, the evaluation of both the IBS and C-index points to model M4 as the model with the highest predictive ability on the GDC data set.

Again, we look at the differences between the purely clinical and the combined models for each level of clinical information in order to assess the added predictive value of the omics data. Once more, the differences for this measure are on a considerably smaller scale than for the Hatzis data set and 0 is always included in the inter-quartile range. From Fig. 4d we note that there are also negative differences for the C-index values. This supports the results obtained from the IBS values, that the incorporation of omics variables can lead to overfitting and therefore a lower predictive ability. In addition, the range of differences between the purely clinical and combined models seems to decrease as more clinical information is included in the modeling process. Especially for model M4, the differences seem to vanish. This suggests that, as more clinical information is included, the incorporation of omics variables hardly makes a difference in terms of prediction accuracy. Again, Additional file 1: Section C includes a table to summarize these results on the GDC data set.

**Fig. 5:**
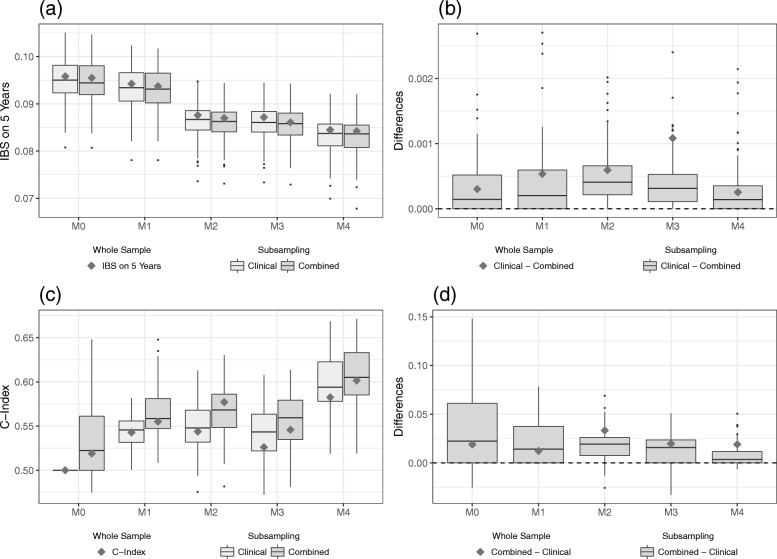
Results on the training part of GDC data. IBS values (**a**) for the clinical and the combined models and the differences (**b**) between the models as evaluated on the training data set. C-index values (**c**) for the clinical and the combined models and the differences (**d**) between the models as evaluated on the training data set. The gray squares indicate the values of the models developed on the whole training data set, whereas the boxplots indicate the values obtained from the subsampling analysis

When evaluation is performed using the training data, the GDC data show a clear downwards trend for the IBS values of both clinical and combined models, see Fig. 5a. Just like on the validation data set, the clinical variables seem to have a higher influence on the predictive ability of the model than the gene expression variables. As a result, we find a rather constant added predictive value of the omics variables for the GDC data (Fig. 5b). This reaffirms that the predictive ability of the gene expressions in the GDC data set, as measured by the IBS, might be rather poor. The results for the C-index, as depicted in Fig. 5c, show a similar picture than on the validation data. While the values for the purely clinical models M1 to M3 seem quite stable, model M4 results in an additional increase in the C-index. For all models considered, however, incorporating omics information raises the apparent predictive ability. The differences between the clinical and the combined models show an apparent added predictive value of the omics data that decreases with the amount of clinical information considered (Fig. 5d). Note that we observe negative differences when evaluating the C-index on the training data, which further corroborates the supposition of little predictive ability of the omics variables on the GDC data set.

### Model complexity

**Fig. 6:**
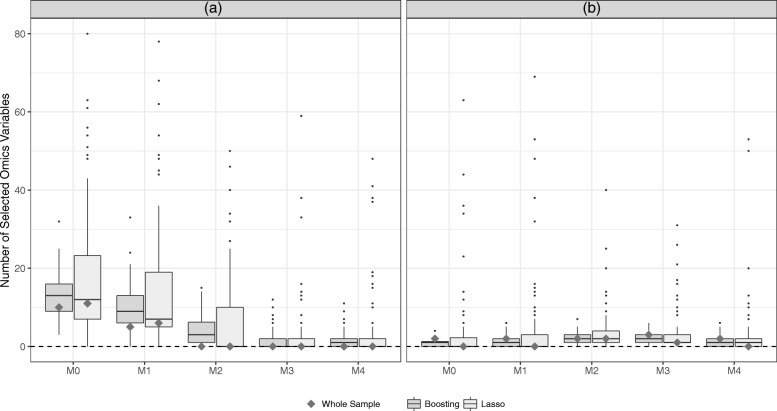
Number of selected omics variables. The number of omics variables included in the different combined models as selected by the boosting (dark gray) and the LASSO (light gray) are compared for the Hatzis (**a**) and the GDC (**b**) data. The gray squares indicate the number of selected omics variables for the models developed on the whole training data set, whereas the boxplots indicate the subsampling analysis

Figure 6 gives some further insight into the complexity of the combined models for all the different levels of clinical information. It is clear that on the Hatzis data (Fig. 6a) both variable selection procedures choose a considerable amount of omics variables for combined models with low levels of clinical information. As the amount of clinical information increases, however, the number of selected omics variables diminishes. In fact, starting with model M2, no omics variables are selected for models developed on the whole data set. We find that, for little clinical information, the LASSO seems to include more omics variables in the modeling process than model based boosting. On the GDC data (Fig. 6b), however, a small number of omics variables are selected, regardless of the amount of clinical information. Here, boosting tends to select at least some omics variables, while the LASSO would not include any predictors other than the clinical information. Combining these findings with the results of the model evaluation, we find that including more clinical information can lead not only to better predictive ability but also to sparser models, in a sense that less omics predictors are identified by the variable selection procedures.

## Discussion

We investigate the added predictive value of gene expression data on two different breast cancer data sets. In cases where we find a noticeable added predictive value, this observation is limited to scenarios with little to no adjustment for already existing clinical predictors. We conclude that for the analyses conducted in this paper, it would have been negligent to disregard the clinical information. The models that show the highest predictive ability on independent data sets include a fair amount of clinical information. At the same time, these models tend to include fewer omics predictors. From a medical point of view, this is somewhat desirable as the collection of omics data is typically more complex than that of clinical data.

However, we want to point out that these results have been obtained from merely two separate data sets, and that in one of them omics data seem to have no prognostic information. It is evident that we cannot sufficiently generalize (or infer), from the two analyzed data sets, to the large variety of omics data. See the recent discussion of comparison studies with data sets playing the role of observations drawn from a fictive population of data sets [36]. Omics data sets are typically very different from one another for various reasons (e.g., technical issues related to the collection of the data, study design, applied preprocessing steps), and yield very variable results due to their small *n*/*p* ratio. In this analysis, we find that the obtained results are highly susceptible to the *researchers’ degrees of freedom*, i.e. to the choices that a data analyst has to make regarding all steps of data analysis; see an introduction to this problem in the seminal work by Simmons et al. [37] and the recent discussion of its consequences in high-dimensional settings [38]. The impact of decisions regarding the analysis strategy can be illustrated by contrasting our results with those of De Bin et al. [6]. Even though one of the analyzed data sets is identical in both studies, there are (minor) discrepancies between the results of the two studies due to differences in study design and implementation details. For example, it is well-known that the results of LASSO depend on the number of variables [39], so it is most likely that the pre-selection step (not performed in De Bin et al. [6]) changes the results noticeably. Other discrepancies may be related to the use of a FP function to model age and to differences in the standardization step when performing boosting/lasso (in De Bin et al. [6] the standardization was performed on the whole training set, while we standardized the variables on the single subsamples).

Furthermore, we want to highlight the importance of the preprocessing steps of omics data. With omics data being such a rich and complex source of information - but also of noise - one might be tempted to readjust and adapt one’s data preprocessing strategy to produce the most interesting or promising results for the analysis. This phenomenon can be seen as just another example of data dredging. To date, no clear and universal consensus has been reached in the scientific community regarding how to best preprocess gene expression data as used in this study. We have therefore made the decision to use the preprocessed form in which the data was made publicly available and to abstain from further preprocessing steps as much as possible. We hope that this approach assures better comparability and generalization. We also want to point out that both data sets have different underlying preprocessing schemes. Both supplied data sources give detailed and precise information on all preprocessing and normalization strategies applied to the data and we would like to refer the interested reader to these extensive resources.

Of course, we do not postulate that gene expression cannot improve the prognostic ability of survival prediction for breast cancer patients. After all, we have analyzed only two data sets that are fairly small and heterogeneous. However, we want to stress the importance of incorporating existing medical knowledge in the model building process as this could possibly have a greater influence than all the tweaking and fine tuning of the methods for the omics data. This proposition is likely to generalize to other fields of medical research and is not specific to the area of breast cancer research.

For many diseases, the literature about prognostic models is confusing and researchers have much freedom to select “their” clinical model. In contrast, in breast cancer we have the NPI as a simple and well established predictor and we agree with the statement from Winzer et al. [11] that the NPI, or their derived extended version NPIext, “can be used to summarize standard clinical information, also serving as a benchmark to assess the added value of new clinical or molecular markers in single studies as well as in the assessment of a marker or a genomic signature in meta-analysis”.

Through our study we also demonstrated the use of two simple well-known regression-based approaches—LASSO and boosting—for this purpose. The idea is to consider the clinical model (with any level of complexity) as an offset when fitting the omics model, which amounts to considering the residuals of the clinical model as a dependent variable. One drawback of this approach is that the clinical model does not adapt to information provided by the omics data. In this study, however, the simplicity in implementation and comparability outweigh this downside. For an extensive discussion of different strategies to combine clinical and omics data see, for example, [21]. Furthermore, we conducted the same analyses on a large number of subsamples drawn randomly from the original data, an increasingly used approach which allows us to investigate the stability of results [40, 41]. This procedure may be used in future research when evaluating the added predictive value of omics data.

Moreover, our study again emphasizes the need for independent evaluation of models. When working with complex omics data it is crucial not to lose track of basic statistical modeling principles, in particular to care about overfitting issues when reporting prediction accuracy. However, this recommendation is not specific to the omics part of the predictor: we observe that the clinical data can also be susceptible to overfitting. Thus, when incorporating clinical information into the analysis, it remains of utmost importance to follow good statistical practice and evaluate the models on independent data sets. Even though the message we want to convey with this project might lead to more complex and time consuming analyses, we assume that this will also lead to more reliable research.

It would be of interest to apply the same principle of the study design to more data sets and different fields of medical research. In our experience it is unfortunately still not common to publish omics data together with a decent amount of well-documented curated clinical information. Of course, there are a few noteworthy exceptions. We hope that through our study we can emphasize the importance of clinical variables for survival prediction and highlight the need for better scientific practice and publication standards in this respect.

## Conclusions

This study uses two exemplary data sets in the field of survival prognosis for breast cancer patients to highlight the importance of incorporating all available clinical information in omics research. The apparent added predictive value of omics data is susceptible to the degree to which the clinical data is exploited in the analysis. We, therefore, advocate a better use of standard clinical data in omics research. Simple and straightforward procedures can be applied to appropriately consider common medical knowledge.

## Additional files

Additional file 1 This.pdf contains a short description of the GDC data set as well as some additional results. (PDF 208 kb)

Additional file 2 This.zip file contains all the codes necessary to reproduce the analysis presented in this study. (ZIP 21 kb)

## Data Availability

The Hatzis data that support the findings of the current study are available in the GEO database under the accession number GSE25066, https://www.ncbi.nlm.nih.gov/geo/query/acc.cgi?acc=GSE25066. The GDC data that support the findings of this study are available from Genomics Data Commons Data Portal, https://portal.gdc.cancer.gov/. Our analyses performed with R can be exactly reproduced by mouse-click using the codes available from Additional file 2.

## Abbreviations

C-index: Concordance index
FP: Fractional polynomials
GDC: Genomic data commons
GEO: Gene expression omnibus
IBS: Integrated Brier score
LASSO: Least absolute shrinkage and selection operator
NPI: Nottingham prognostic index
TCGA: The cancer genome atlas

## Footnotes

1 In this paper, we primarily use the term “predictive” when talking about a model’s ability to predict the outcome even though the model itself might be considered “prognostic”, i.e. it is concerned with the outcome, independent of the treatment received.

## References

[1] Ioannidis JP (2005). Microarrays and molecular research: noise discovery?. Lancet.

[2] Ioannidis JP, Greenland S, Hlatky MA, Khoury MJ, Macleod MR, Moher D, Schulz KF, Tibshirani R (2014). Increasing value and reducing waste in research design, conduct, and analysis. Lancet.

[3] Yuan Y, Van Allen EM, Omberg L, Wagle N, Amin-Mansour A, Sokolov A, Byers LA, Xu Y, Hess KR, Diao L (2014). Assessing the clinical utility of cancer genomic and proteomic data across tumor types. Nat Biotechnol.

[4] Binder H, Schumacher M (2008). Allowing for mandatory covariates in boosting estimation of sparse high-dimensional survival models. BMC Bioinformatics.

[5] Bøvelstad HM, Nygård S, Borgan Ø (2009). Survival prediction from clinico-genomic models-a comparative study. BMC Bioinformatics.

[6] De Bin R, Sauerbrei W, Boulesteix A-L (2014). Investigating the prediction ability of survival models based on both clinical and omics data: two case studies. Stat Med.

[7] Vazquez AI, Veturi Y, Behring M, Shrestha S, Kirst M, Resende MF, de los Campos G (2016). Increased proportion of variance explained and prediction accuracy of survival of breast cancer patients with use of whole-genome multiomic profiles. Genetics.

[8] Dimitrieva S, Schlapbach R, Rehrauer H (2016). Prognostic value of cross-omics screening for kidney clear cell renal cancer survival. Biol Direct.

[9] Haybittle J, Blamey R, Elston C, Johnson J, Doyle P, Campbell F, Nicholson R, Griffiths K (1982). A prognostic index in primary breast cancer. Br J Cancer.

[10] Blamey R, Ellis I, Pinder S, Lee A, Macmillan R, Morgan D, Robertson J, Mitchell M, Ball G, Haybittle J (2007). Survival of invasive breast cancer according to the Nottingham Prognostic Index in cases diagnosed in 1990–1999. Eur J Cancer.

[11] Winzer K-J, Buchholz A, Schumacher M, Sauerbrei W (2016). Improving the prognostic ability through better use of standard clinical data-the Nottingham Prognostic Index as an example. PLoS ONE.

[12] Cox DR (1972). Regression models and life-tables. J R Stat Soc Ser B Methodol.

[13] Hatzis C, Pusztai L, Valero V, Booser DJ, Esserman L, Lluch A, Vidaurre T, Holmes F, Souchon E, Wang H (2011). A genomic predictor of response and survival following taxane-anthracycline chemotherapy for invasive breast cancer. J Am Med Assoc.

[14] Barrett T, Wilhite SE, Ledoux P, Evangelista C, Kim IF, Tomashevsky M, Marshall KA, Phillippy KH, Sherman PM, Holko M, Yefanov A, Lee H, Zhang N, Robertson CL, Serova N, Davis S, Soboleva A (2013). NCBI GEO: archive for functional genomics data sets—update. Nucleic Acids Res.

[15] Buuren S. v., Groothuis-Oudshoorn K (2011). mice: Multivariate imputation by chained equations in R. J Stat Softw.

[16] Royston P, Sauerbrei W (2008). Multivariable Model-Building: A Pragmatic Approach to Regression Analysis Based on Fractional Polynomials for Modelling Continuous Variables. Wiley Series in Probability and Statistics.

[17] Benner A (2005). mfp: Multivariable fractional polynomials. R News.

[18] Grossman RL, Heath AP, Ferretti V, Varmus HE, Lowy DR, Kibbe WA, Staudt LM (2016). Toward a shared vision for cancer genomic data. N Engl J Med.

[19] Cancer Genome Atlas Network (2012). Comprehensive molecular portraits of human breast tumours. Nature.

[20] Breiman L (2001). Random forests. Mach Learn.

[21] Boulesteix A-L, Sauerbrei W (2011). Added predictive value of high-throughput molecular data to clinical data and its validation. Brief Bioinform.

[22] Tibshirani R (1997). The lasso method for variable selection in the Cox model. Stat Med.

[23] Bühlmann P, Yu B (2003). Boosting with the L _2_ loss: regression and classification. J Am Stat Assoc.

[24] De Bin R (2016). Boosting in Cox regression: a comparison between the likelihood-based and the model-based approaches with focus on the R-packages CoxBoost and mboost. Comput Stat.

[25] Friedman J, Hastie T, Tibshirani R (2010). Regularization paths for generalized linear models via coordinate descent. J Stat Softw.

[26] Hothorn T, Bühlmann P, Kneib T, Schmid M, Hofner B (2010). Model-based boosting 2.0. J Mach Learn Res.

[27] Royston P, Altman DG (2013). External validation of a Cox prognostic model: principles and methods. BMC Med Res Methodol.

[28] Rahman MS, Ambler G, Choodari-Oskooei B, Omar RZ (2017). Review and evaluation of performance measures for survival prediction models in external validation settings. BMC Med Res Methodol.

[29] Cook NR (2018). Quantifying the added value of new biomarkers: how and how not. Diagn Prognostic Res.

[30] Harrell FE, Lee KL, Mark DB (1996). Multivariable prognostic models: issues in developing models, evaluating assumptions and adequacy, and measuring and reducing errors. Stat Med.

[31] Gerds TA, Kattan MW, Schumacher M, Yu C (2013). Estimating a time-dependent concordance index for survival prediction models with covariate dependent censoring. Stat Med.

[32] Graf E, Schmoor C, Sauerbrei W, Schumacher M (1999). Assessment and comparison of prognostic classification schemes for survival data. Stat Med.

[33] Schumacher M, Binder H, Gerds T (2007). Assessment of survival prediction models based on microarray data. Bioinformatics.

[34] De Bin R, Herold T, Boulesteix A-L (2014). Added predictive value of omics data: specific issues related to validation illustrated by two case studies. BMC Med Res Methodol.

[35] Mogensen UB, Ishwaran H, Gerds TA (2012). Evaluating random forests for survival analysis using prediction error curves. J Stat Softw.

[36] Boulesteix A-L, Wilson R, Hapfelmeier A (2017). Towards evidence-based computational statistics: lessons from clinical research on the role and design of real-data benchmark studies. BMC Med Res Methodol.

[37] Simmons JP, Nelson LD, Simonsohn U (2011). False-positive psychology: Undisclosed flexibility in data collection and analysis allows presenting anything as significant. Psychol Sci.

[38] Boulesteix A-L, Hornung R, Sauerbrei W, Pietsch W, Wernecke J, Ott M (2017). On fishing for significance and statistician’s degree of freedom in the era of big molecular data. Berechenbarkeit der Welt?Philosophie und Wissenschaft Im Zeitalter Von Big Data.

[39] Flynn CJ, Hurvich CM, Simonoff JS (2017). On the sensitivity of the Lasso to the number of predictor variables. Stat Sci.

[40] Meinshausen N, Bühlmann P. Journal of the Royal Statistical Society: Series B (Statistical Methodology). 2010; 72(4):417–73.

[41] Sauerbrei W, Buchholz A, Boulesteix A-L, Binder H (2015). On stability issues in deriving multivariable regression models. Biom J.

