# Supplementary material for: A plea for taking all available clinical information into account when assessing the predictive value of omics data
Source: BMC Med Res Methodol. 2019 Jul 24;19:162. doi: 10.1186/s12874-019-0802-0 (PMC6657034; doi:10.1186/s12874-019-0802-0)
Supplement: Supplementary file 1 — This.pdf contains a short description of the GDC data set as well as some additional results. (PDF 208 kb) [file 12874_2019_802_MOESM1_ESM.pdf]

Online Appendix for "A plea for taking all available clinical information into account when assessing the predictive value of omics data"

Volkmann A., De Bin R., Sauerbrei W., Boulesteix A.-L.

June 12, 2019

Abstract

The online appendix is divided into three sections. First, we present a short description of the GDC data set used in the main analysis. We also show a Kaplan-Meier plot of the underlying data. Second, we describe the results obtained from applying the LASSO instead of the boosting approach. These additional results support the findings of the main analysis presented in the article. Finally, we present tables that summarize the results of the evaluation on the validation data sets of the boosting approach presented in the main analysis.

A GDC Data Set Description

Table 1 gives an overview of the GDC data set used in the analysis. It contains information on the disease free survival outcome of the patients as well as their baseline characteristics. Note that here, we describe the final data set that is used for the model building, i.e. the corresponding preprocessing steps as described in the article have been applied.

Kaplan-Meier estimates for the disease-free survival of the patients in the training and validation part of the GDC data set are given in Figure 1. The estimates show that with respect to disease free survival, the training and validation parts of the GDC data set are comparable.

Table 1: Outcome information and baseline characteristics for the training and validation parts of the GDC data set.

Variable	Training Data	Validation Data
Disease free survival		
Yes	570	286
No	122	61
Nodal status		
N0	338	171
N1	235	114
N2	74	38
N3	45	24
Tumor size		
T0_1	187	85
T2	389	214
T3	94	41
T4	22	7
Estrogen receptor		
Yes	535	257
No	157	90
Age		
Minimum	27	26
Median	59	58
Mean	58.8	57.4
Maximum	90	90

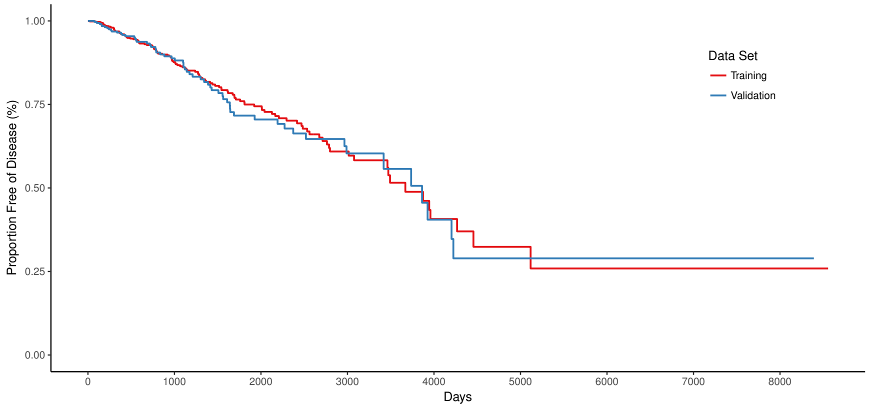

Training	692	299	133	50	15	5	3	3	1
Validation	347	142	59	28	7	3	2	2	2

Numbers at risk

Figure 1: Kaplan-Meier estimates of the disease free survival time for patients of the training and validation parts of the GDC data set. The numbers of patients at risk for each time point are also given.

B LASSO Results

Overall, the results of the LASSO approach when selecting the omics variables are very similar to the results of the boosting approach presented in the article. However, the analysis using the LASSO takes longer for the implementation used in this paper. The results are presented for each data set separately.

B.1 Hatzis Data Set

Figure 2 (A) shows the IBS values for the models derived from the whole or subsampled training data sets when evaluated on the validation part of the Hatzis data. Again, we see a slight upward trend for the models M0-M1, then a downward trend for models M1-M3 and signs of overfitting for model M4. The differences between the purely clinical an combined models in Figure 2 (B) thus also show a diminishing added predictive value of the omics variables for models including more clinical information in the modeling process. Note that here, we see more negative values than in the corresponding plot of the boosting approach. This suggests that the omics variables identified by the LASSO might be more susceptible to overfitting. It is also apparent that for models M3 and M4, the LASSO does not select any omics variables into the combined models as most of the differences are zero. The best overall model as evaluated using the IBS seems to be model M3.

Figure 3 (A) gives the C-index values for the clinical and combined models as evaluated on the validation part of the Hatzis data. For the clinical models we discern an upward trend for M0-M3 with M4 showing signs of overfitting. On all levels of clinical information, the C-index values seem to be higher for the combined models, which is especially true for the models with little clinical information (M0 and M1). Again, the combined model for M0 seems to yield the best results on the validation data. Consequently, for the differences presented in Figure 3 (B), we see a diminishing added predictive value for the increasing amounts of clinical information.

When evaluated on the training data, the IBS values (Figure 4 (A)) show a downward trend for the clinical but not for the combined models. This results in a diminishing added predictive value when more clinical information is included into the modeling process (Figure 4 (B)). For the C-index values depicted in Figure 4 (C), we again find that there are big differences between the purely clinical and the combined models for low levels of clinical information but not for higher amounts of clinical information. Similar to the boosting scenario, the highest C-index values for the evaluation on the training data are obtained for the combined model M0 where no clinical information is taken into account. The differences between the two models again show a downward trend (Figure 4 (D)).

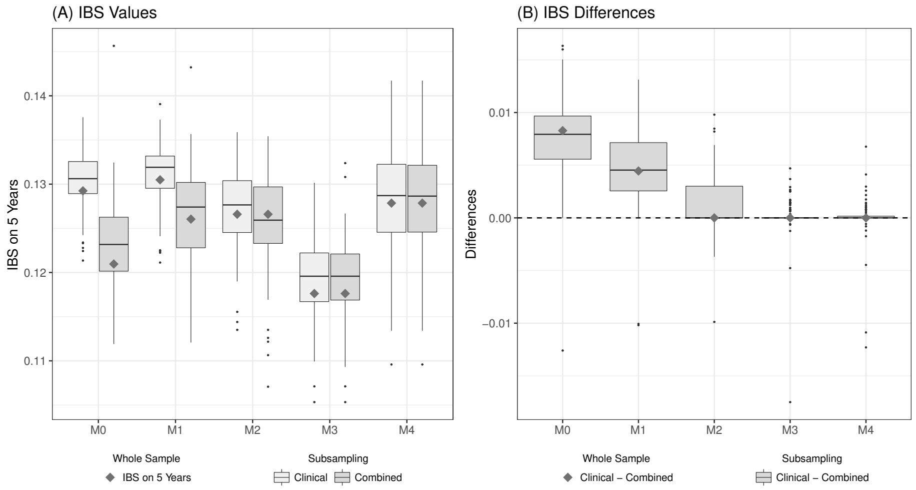

Figure 2: Hatzis data: IBS values (A) for the clinical and the combined models and the differences (B) between the models as evaluated on the validation data set. The black diamonds indicate the values of the models developed on the whole training data set whereas the boxplots indicate the values of the subsampling scheme.

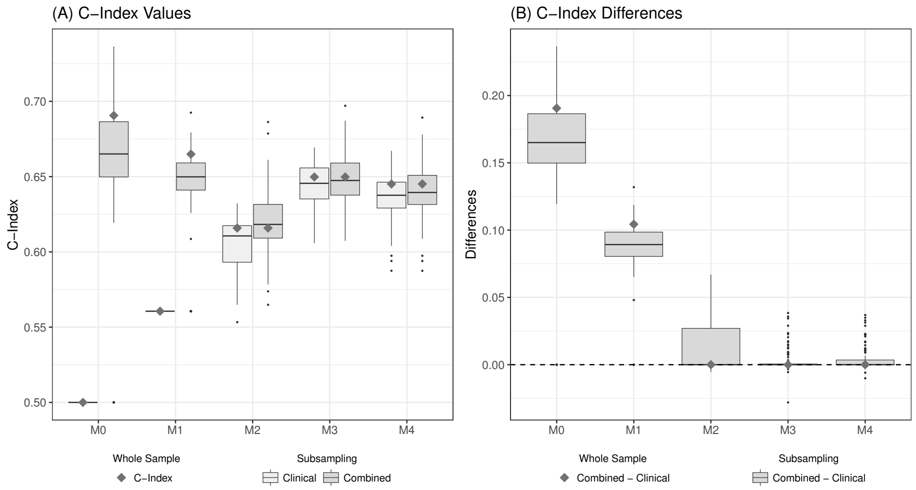

Figure 3: Hatzis data: C-index values (A) for the clinical and the combined models and the differences (B) between the models as evaluated on the validation data set. The black diamonds indicate the values of the models developed on the whole training data set whereas the boxplots indicate the values of the subsampling scheme.

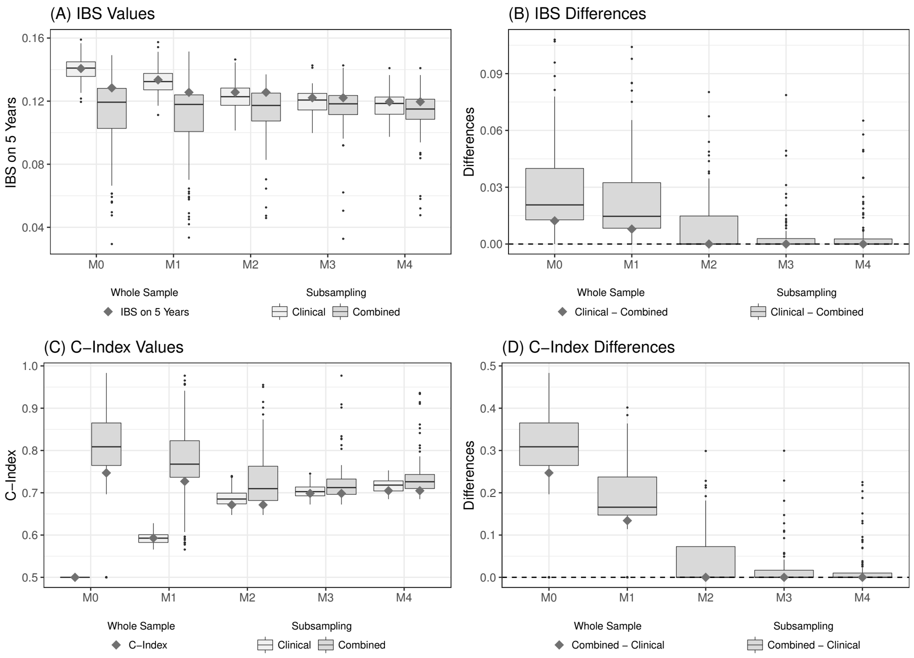

Figure 4: Hatzis data: IBS values (A) for the clinical and the combined models and the differences (B) between the models as evaluated on the training data set. C-index values (C) for the clinical and the combined models and the differences (D) between the models as evaluated on the training data set. The black diamonds indicate the values of the models developed on the whole training data set whereas the boxplots indicate the values obtained from the subsampling analyses.

B.2 GDC Data Set

Figure 5 (A) shows the IBS values derived from the whole and subsampled training part of the GDC data. Two downward trends can be discerned (M0-M1, M2-M4) with M4 yielding the overall best models. However, it is apparent that the combined models tend to yield higher values. This can be confirmed by looking at the IBS differences in Figure 5 (B). Compared to the boosting approach presented in the article, we see more pronounced negative differences implying that the selected omics variables do not improve the predictive ability but lead to overfitting.

Evaluating the C-index on the validation part of the GDC data yields Figure 6 (A). Model M0 is on a lower overall level than the models M1-M3 with M4 yielding the highest C-index values. Note that for the combined models M0 and M1, the selection procedure often does not include any omics variables in the final model (indicated by the narrow boxplots). The differences depicted in Figure 6 (B) show that the combined models often yield a negative difference meaning that the predictive ability of the models deteriorates by including gene expression variables when evaluated on the validation data set.

When evaluated on the training data, the IBS values (Figure 7 (A)) show the expected clear downward trend for models with more clinical information. When the differences between the clinical and the combined models are considered (Figure 7 (B)) we also see that often, no omics variables are selected. The differences are positive but diminish for higher levels of clinical information. Figure 7 (C) shows the C-index values for the whole and subsampled training data evaluated on the training data. A slight upward trend can be discerned with M4 again yielding the highest overall values. The differences between the models (Figure 7 (D)) show (mainly) positive values with a lower range for models with higher amount of clinical information.

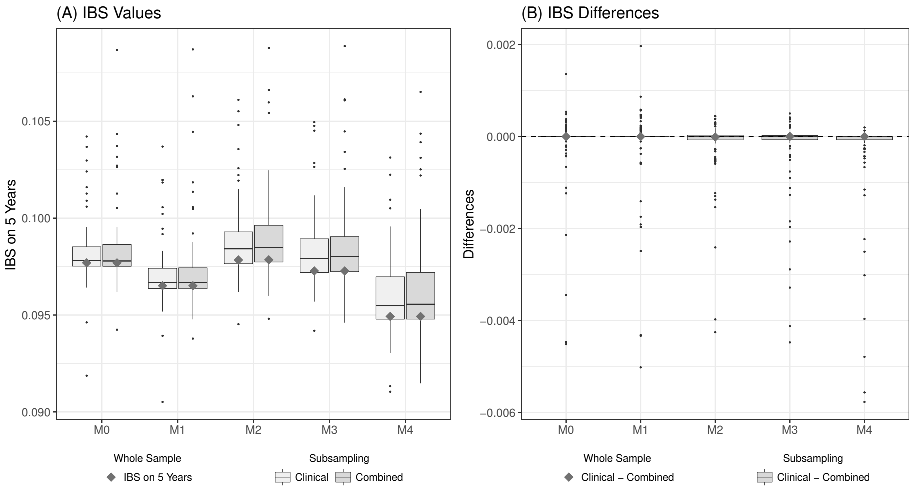

Figure 5: <u>GDC data:</u> IBS values (A) for the clinical and the combined models and the differences (B) between the models as evaluated on the validation data set. The black diamonds indicate the values of the models developed on the whole training data set whereas the boxplots indicate the values obtained from the subsampling analyses.

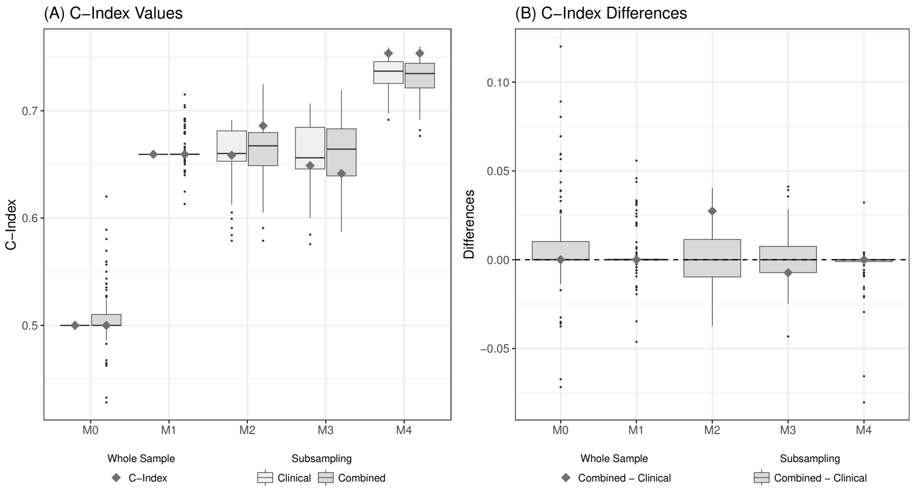

Figure 6: <u>GDC data:</u> C-index values (A) for the clinical and the combined models and the differences (B) between the models as evaluated on the validation data set. The black diamonds indicate the values of the models developed on the whole training data set whereas the boxplots indicate the values obtained from the subsampling analyses.

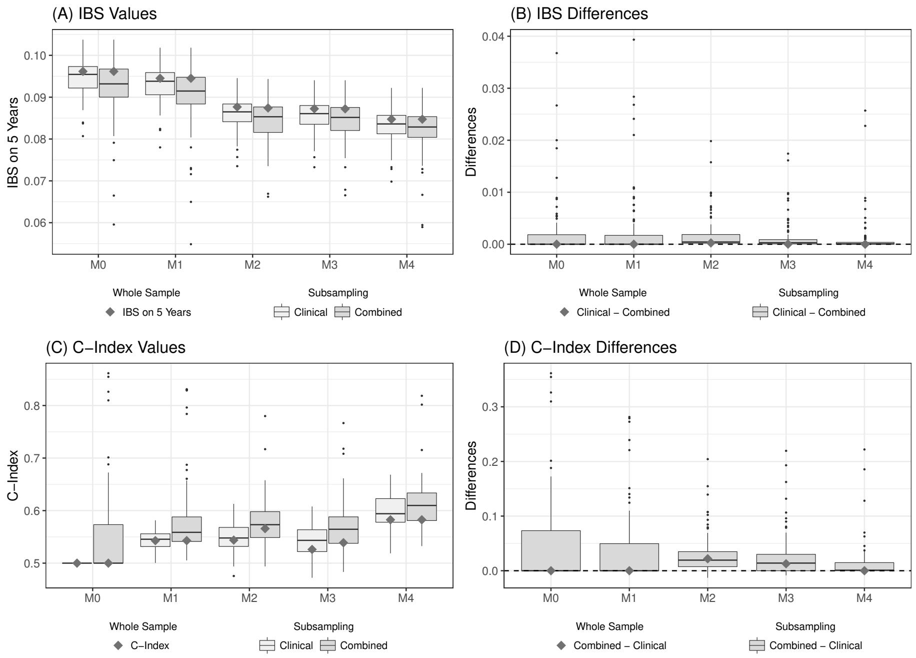

Figure 7: <u>GDC data:</u> IBS values (A) for the clinical and the combined models and the differences (B) between the models as evaluated on the training data set. C-index values (C) for the clinical and the combined models and the differences (D) between the models as evaluated on the training data set. The black diamonds indicate the values of the models developed on the whole training data set whereas the boxplots indicate the values obtained from the subsampling analyses.

C Summary of Boosting Results

Tables 2 and 3 give an overview of the results obtained with the Hatzis and GDC data set, respectively, when evaluating the clinical and combined models derived from the whole sample and within the subsampling scheme (i.e. with subsamples randomly drawn from the training data set) on the validation data. The subsampling results are summarized using the median value for both IBS and C-index.

Table 2: Results on Hatzis data. The table contains the values of the IBS and C-index of the models derived from the whole training data set and the median values of the subsampling scheme. In both cases, the models are evaluated on the validation data. The asterisk indicates that the value 0.5 (corresponding to random guess) is set without computation.

Model	IBS				C-index			
	Whole Sample		Subsampling		Whole Sample		Subsampling	
	Clinical	Combined	Clinical	Combined	Clinical	Combined	Clinical	Combined
M0	0.129	0.120	0.131	0.122	0.5*	0.690	0.5*	0.679
M1	0.130	0.125	0.132	0.127	0.561	0.663	0.561	0.653
M2	0.126	0.125	0.128	0.127	0.616	0.627	0.611	0.622
M3	0.117	0.117	0.120	0.120	0.650	0.651	0.646	0.651
M4	0.127	0.127	0.129	0.129	0.645	0.647	0.638	0.640

Table 3: Results on GDC data. The table contains the values of the IBS and C-index of the models derived from the whole training data set and the median values of the subsampling scheme. In both cases, the models are evaluated on the validation data. The asterisk indicates that the value 0.5 (corresponding to random guess) is set without computation.

Model	IBS				C-index			
	Whole Sample		Subsampling		Whole Sample		Subsampling	
	Clinical	Combined	Clinical	Combined	Clinical	Combined	Clinical	Combined
M0	0.098	0.098	0.098	0.098	0.5*	0.550	0.5*	0.506
M1	0.097	0.096	0.097	0.097	0.659	0.686	0.659	0.662
M2	0.098	0.098	0.098	0.098	0.659	0.684	0.660	0.668
M3	0.097	0.097	0.098	0.098	0.649	0.700	0.656	0.667
M4	0.095	0.095	0.096	0.096	0.754	0.755	0.737	0.737
